# Amitriptyline potently neutralizes distinct SARS-CoV-2 variants including D614G, Omicron BA.5, and Omicron XBB.1

**DOI:** 10.3389/fmicb.2025.1631947

**Published:** 2025-08-04

**Authors:** Isabel Zydek, Laura Thümmler, Nadine Beckmann, Carolin Sehl, Matthias Soddemann, Rabea Grüneberg, Carina Elsner, Markus Kamler, Ulf Dittmer, Oliver Witzke, Stephanie Kadow, Erich Gulbins, Katrin Anne Becker, Adalbert Krawczyk

**Affiliations:** ^1^Department of Infectious Diseases, West German Centre of Infectious Diseases, University Medicine Essen, University Hospital Essen, University Duisburg-Essen, Essen, Germany; ^2^Institute of Molecular Biology, University Hospital Essen, University of Duisburg-Essen, Essen, Germany; ^3^Institute for Virology, University Hospital Essen, University Duisburg-Essen, Essen, Germany; ^4^Department of Thoracic and Cardiovascular Surgery, West German Heart Center, University Hospital Essen, Essen, Germany

**Keywords:** SARS-CoV-2, antidepressants, amitriptyline, COVID-19, FIASMA

## Abstract

**Introduction:**

COVID-19, caused by SARS-CoV-2, remains a global health challenge despite the availability of vaccines and antiviral treatments. The emergence of immune escape variants and the persistence of long COVID symptoms continue to complicate prevention and therapy, especially in low-resource settings. Functional inhibitors of acid sphingomyelinase (FIASMAs) have shown promise as broad-spectrum antiviral agents.

**Methods:**

We evaluated the antiviral efficacy of amitriptyline, a widely used antidepressant and known FIASMA, against SARS-CoV-2. In vitro neutralization assays using pseudotyped virus-like particles (VLPs) and clinical isolates were performed to assess its ability to inhibit viral entry and replication.

**Results:**

Amitriptyline significantly inhibited infection by SARS-CoV-2 VLPs bearing spike proteins, including those with mutations in the receptor-binding domain. Moreover, it reduced replication of clinical SARS-CoV-2 isolates D614G, Omicron BA.5, and Omicron XBB.1 in a dose-dependent manner at subtoxic concentrations.

**Discussion:**

Our findings demonstrate that amitriptyline neutralizes SARS-CoV-2 across multiple variants. These results support the potential of amitriptyline as a repurposed antiviral drug. Further clinical studies are warranted to evaluate its efficacy and safety in treating COVID-19 in humans.

## 1 Introduction

Since the emergence of SARS-CoV-2, the global infection landscape has shifted from a pandemic to an endemic state (Antia and Halloran, 2021; Veldhoen and Simas, 2021). Nevertheless, SARS-CoV-2 infections remain a significant public health concern (Cascella et al., 2025). An additional challenge arises from the virus’s mutations. These often render neutralizing antibodies induced by vaccinations or previous infections, insufficient to protect against newly emerging variants (Machkovech et al., 2024). In particular, mutations in the receptor-binding domain (RBD) of the SARS-CoV-2 spike protein (e.g., N501Y in Alpha, E484K in Beta/Gamma, L452R/T478K in Delta, and K417N/T478K/N501Y in Omicron) led to the emergence of new variants against which the initially approved vaccines have significantly lost effectiveness (Harvey et al., 2021; Mengist et al., 2021). Even a previous infection no longer provided sufficient protection against reinfection with the new SARS-CoV-2 variants, such as Omicron BA.5, and Omicron XBB.1 (Hachmann et al., 2022; Yang et al., 2023). The rising incidence of breakthrough infections highlighted the need for vaccines to be adjusted accordingly (Hacisuleyman et al., 2021; Bormann et al., 2023; Zou et al., 2023). The BioNTech/Pfizer vaccine adapted to the Omicron variant XBB.1.5 was authorized by the European Commission and the Food and Drug Administration (FDA) in September, 2023 (Lin et al., 2024). The vaccine was specifically developed for the autumn and winter season to provide protection against XBB sublineages such as XBB.1.5, XBB.1.16, and EG.5.1 (Lin et al., 2024). It is, however, expected that the COVID-19 vaccine will need to be continuously updated to provide sufficient protection against newly emerging variants (Raharinirina et al., 2025). Currently authorized small-molecule antivirals for the treatment of COVID-19 include nirmatrelvir/ritonavir (Paxlovid), molnupiravir, and remdesivir. While these agents have shown clinical benefit in early infection, they must be administered shortly after symptom onset, and their efficacy may be reduced against emerging variants or in immunocompromised individuals (Jayk Bernal et al., 2022). Moreover, recent reports have raised concerns about the development of viral resistance, especially with protease inhibitors such as nirmatrelvir (Yaghi et al., 2024). These limitations underline the continued need for broadly acting antiviral strategies that are less susceptible to viral escape mutations. Similarly, the situation is challenging for antiviral drugs against SARS-CoV-2. In particular, monoclonal antibodies, which were successfully used to treat acute SARS-CoV-2 infections, have shown little to no efficacy against the constantly evolving SARS-CoV-2 variants (Chen et al., 2021; Mazzaferri et al., 2022; Murakami et al., 2023).

In this context, there is still a need for variant-independent approaches for the prevention and treatment of SARS-CoV-2 infections (Sahebkar et al., 2023; Batool et al., 2025). Obviously, targeting a mechanism other than the binding of the receptor-binding domain (RBD) to the cellular receptor ACE2 may be less susceptible to escape mutations, enabling potent neutralization of SARS-CoV-2 in a variant-independent manner. The acid sphingomyelinase/ceramide system has been shown to play a crucial role in bacterial and viral infections, including *Pseudomonas aeruginosa*, *Staphylococcus aureus*, Ebola, Rhinoviruses, and SARS-CoV-2 (Grassme et al., 2005; Miller et al., 2012; Kornhuber et al., 2022). The antidepressants fluoxetine, fluvoxamine, amitriptyline and sertraline are known as functional inhibitors of the acid sphingomyelinase (FIASMAs), and some of them were already shown to potently inhibit viral infections, including Zika virus, MERS-CoV and early variants of SARS-CoV-2 (Kornhuber et al., 2022; Kutkat et al., 2022; Zhang et al., 2023). Since antidepressants are already approved and widely available medications worldwide, and patients treated with these antidepressants have experienced milder courses of COVID-19, it makes sense to further investigate their efficacy against various SARS-CoV-2 variants (Lenze et al., 2020; Hoertel et al., 2021; Reis et al., 2022). Several clinical studies have evaluated the effects of amitriptyline or related FIASMAs in COVID-19 patients. For example, fluvoxamine and fluoxetine, both FIASMAs with a similar mechanism of action, have been investigated in randomized controlled trials and observational studies, demonstrating reductions in disease progression, hospitalization rates, and severity of symptoms including respiratory distress and systemic inflammation (Lenze et al., 2020; Hoertel et al., 2021; Reis et al., 2022). While amitriptyline itself has not yet been extensively trialed in large COVID-19 cohorts, retrospective data from patients treated with tricyclic antidepressants suggest a trend toward milder COVID-19 courses and improved outcomes (Hoertel et al., 2021). These findings support further exploration of amitriptyline as a clinically relevant candidate, especially given its established safety profile and pharmacological characteristics. Among these, amitriptyline possesses several features that underscore its clinical relevance in the context of SARS-CoV-2. It is a well-characterized, widely available antidepressant with decades of clinical use and a favorable safety profile (Gillman, 2007; Kornhuber et al., 2022). Its high oral bioavailability, extensive tissue distribution, and strong blood–brain barrier penetration may be beneficial, particularly in light of the neurological complications observed in COVID-19 patients (Ellul et al., 2020). Moreover, the pharmacokinetic profile of amitriptyline, including its high oral bioavailability, well-characterized metabolism, and elimination kinetics, is well established. This thorough pharmacological understanding supports its potential for repurposing in diverse clinical settings, where predictable drug behavior and safety are essential (Gillman, 2007). Given these favorable pharmacological properties and its established clinical profile, amitriptyline emerges as a promising candidate for therapeutic repurposing in the context of COVID-19. This provided the rationale for investigating its potential antiviral activity *in vitro*. In this study, we evaluated the antiviral efficacy of amitriptyline against distinct SARS-CoV-2 clinical isolates, including D614G, Omicron BA.5, and Omicron XBB.1.

## 2 Materials and methods

### 2.1 Cells and viruses

All cell lines were incubated in a standard tissue incubator at 37°C and 5% CO_2_. For the production of VSV*ΔG-FLuc-SARS-CoV-2 spike-pseudotyped virus-like particles (pp-VSV-SARS-CoV-2 spike), HEK293T (human kidney cell line) cells were used. HEK293T cells were purchased from Merck (Darmstadt, Germany) and maintained in Dulbecco’s modified Eagle medium (DMEM; Thermo Fisher Scientific, Waltham, MA, United States) with 10% fetal bovine serum (FBS) (GE Healthcare, Chicago, IL, United States), penicillin (100 U/mL) and streptomycin (100 μg/mL) (Thermo Fisher Scientific) (Thümmler et al., 2024). For initial infections experiments with pseudotyped virus-like particles, Vero E6 (African green monkey kidney cell line) were used (ATCC CRL-1587). Vero E6 cells were maintained in DMEM supplemented with 10% FBS, penicillin (100 U/mL), streptomycin (100 μg/mL), 100 μM non-essential amino acids, 1 mM sodium pyruvate (each Thermo Fisher Scientific) and 10 nM HEPES, pH 7.3 (Carl Roth, Karlsruhe, Germany). A549-AT cells were kindly provided my Marek Widera (Institute of Virology, Frankfurt, Germany) and cultivated in minimum essential medium (MEM) (Gibco, Darmstadt, Germany), supplemented with penicillin (100 U/mL) and streptomycin (100 μg/mL) (Gibco), 10% (v/v) fetal calf serum (FCS) (Biowest, Bradenton, United States) at 37°C with 5% CO_2_. A549-AT overexpress ACE2 and TMPRSS2 and were genetically engineered to ensure comparable infectivity with different SARS-CoV-2 variants (Widera et al., 2021).

Clinical SARS-CoV-2 isolates D614G, BA.5, and XBB.1 were derived from nasopharyngeal swabs from hospitalized COVID-19 patients, as described before (Heilingloh et al., 2020; Thümmler et al., 2022). SARS-CoV-2 variants were propagated on A549-AT cells and afterwards stored at −80°C. Viral titers were determined by endpoint dilution on A549-AT cells and calculated as 50% tissue culture infective dose (TCID50)/mL as described previously (Bormann et al., 2023). SARS-CoV-2 isolates were sequenced using the EasySeqTM SARS-CoV-2 Whole Genome HGS Sequencing kit (Nimagen, Nijmegen, The Netherlands) as previously described (Bormann et al., 2023; Cherneha et al., 2024). SARS-CoV-2 variants of concern were identified through sequence analysis, utilizing the WHO’s official list of variants (WHO, 2024).

### 2.2 Plasmids

For initial neutralization assays, pp-VSV-SARS-CoV-2 spike were used. Some of these particles were produced using the pCG1_SARS-2-S-del18 plasmid, generously supplied by Markus Hoffmann and Stefan Pöhlmann (Infection Biology Unit, German Primate Centre-Leibniz Institute for Primate Research, Göttingen, Germany) as previously described (Hoffmann et al., 2020). Variants of the SARS-CoV-2 spike protein with specific point mutations in the receptor-binding domain (RBD) were generated using the Q5 site-directed mutagenesis kit (New England Biolabs, Ipswich, MA, United States), following the manufacturer’s guidelines, as described previously (Thümmler et al., 2024). Other particles were generated using expression plasmids containing complete mutations for various variants of concern were kindly provided by David Nemazee (Wt: pCDNA3.3_CoV2_D18, Addgene plasmid #170442; alpha: pCDNA3.3_CoV2_B.1.1.7, Addgene plasmid #170451; beta: pCDNA3.3_CoV2_501V2, Addgene plasmid #170449; gamma: pCDNA3.3_CoV2_P1, Addgene plasmid #170450; delta: pCDNA3.3-SARS2-B.1.617.2, Addgene plasmid #172320) or obtained from GenScript (Omicron: SARS-CoV-2 Omicron Strain S gene Human codon_pcDNA3.1(+), #MC_0101274) (Thümmler et al., 2024). Plasmids were amplified in *E. coli* DH5α competent cells (New England Biolabs) and subsequently isolated and purified using Maxiprep kits according to the manufacturer’s protocols from Qiagen (Hilden, Germany) and Macherey-Nagel (Düren, Germany).

### 2.3 Preparation of VSV-SARS-CoV-2 spike-pseudotyped virus-like particles

The replication-restricted VSV (∆G) system for pseudoviral particles, which includes enhanced green fluorescent protein (eGFP) and firefly luciferase (FLuc) reporters (VSV*∆G-FLuc), was generously provided by Gert Zimmer (Institute of Virology and Immunology, Mittelhäusern, Switzerland), via Stefan Pöhlmanns lab. This system has been previously described by Berger Rentsch and Zimmer (2011). The generation of pseudoviral particles was performed as previously described (Becker et al., 2021; Thümmler et al., 2024). Briefly, VSV*ΔG-FLuc (VSV-G) stocks and anti-VSV-G antibody supernatants were prepared and titrated as described according to the published protocol. The SARS-CoV-2 spike-pseudotyped VSV particles (pp-VSV-SARS-CoV-2 spike) were generated following the described protocol with slight modifications. Low-passage HEK293T cells were seeded into 10 cm cell culture dishes at a density of 1.8 × 10^6^ cells per dish and cultured for 24 h at 37°C with 5% CO_2_ in a standard cell culture incubator. On the following day, the culture medium was refreshed (9 mL per dish), and transfection mixtures were prepared by combining 42 μg of the respective plasmid DNA per dish with sterile ultrapure water to a final volume of 400 μL. Next, 100 μL of sterile-filtered CaCl_2_ (2.5 M stock solution, Thermo Fisher Scientific) was added and mixed thoroughly. Subsequently, 500 μL of 2 × HBS buffer (280 mM NaCl, 50 mM HEPES, 20 mM KCl, 1.5 mM Na_2_HPO_4_, pH 7.1, Thermo Fisher Scientific) was added dropwise while bubbling the solution with an electronic pipettor. The mixture was immediately vortexed and left to incubate at room temperature for 20 min. The transfection complexes were then added dropwise to the cells and incubated for 28–30 h at 33°C with 5% CO_2_. Afterward, cells were inoculated with VSV*ΔG-FLuc at a multiplicity of infection (MOI) of 5 for 1 h at 33°C and 5% CO_2_ in a standard tissue culture incubator. The supernatant was subsequently removed, and cells were washed twice with PBS (Thermo Fisher Scientific). Fresh medium containing anti-VSV-G antibodies was then added, and cells were incubated overnight (18 h) at 33°C with 5% CO_2_. The supernatant containing the pseudoviral particles was collected, cellular debris was removed by centrifugation at 2,000 × g for 10 min, and the clarified supernatant was immediately used for experiments.

### 2.4 Transduction *in vitro* experiments

Vero E6 cells were plated at a density of 1 × 10^4^ cells per well in 48-well plates and cultured for 48 h. Before infection, the cells were pre-treated 4 h with amitriptyline in DMEM (Thermo Fisher Scientific) supplemented with 1% FBS (Thermo Fisher Scientific). Amitriptyline (Sigma-Aldrich, St. Louis, MO, United States) was dissolved in 0.9% NaCl (BRAUN, Melsungen) and diluted to a final concentration of 10 μM. Mock-treated cells were used as controls. After 4 h of incubation, the inoculation medium was removed, and the cells were exposed to pp-VSV-SARS-CoV-2 spike-containing medium with 25 μM amitriptyline. Medium containing pp-VSV-SARS-CoV-2 spike and the corresponding volume of 0.9% NaCl without amitriptyline was used as a control. After infection for 1 h at 33°C and 5% CO_2_, pseudoviral supernatants were discarded, and fresh medium was added, followed by an 18-h incubation at 33°C with 5% CO_2_. The impact of amitriptyline on infection was evaluated through qualitative and quantitative eGFP expression assessments Fluorescence images were acquired using an EVOS microscope (Thermo Fisher Scientific). For quantitative analysis, cells were harvested and analyzed by flow cytometry (Attune NxT, Thermo Fisher Scientific). Infection levels in the mock controls were set to 100%, and the effects of amitriptyline were calculated relative to these controls.

### 2.5 Neutralization assay

The neutralization capacity of amitriptyline against the SARS-CoV-2 variants wild-type (D614G), Omicron BA.5, and Omicron XBB.1 was analyzed using an endpoint dilution assay. A549-AT cells were seeded in a 24-well plate (at 2 × 10^4^ cells per well) and cultured for 24 h at 37°C with 5% CO_2_. Subsequently, the confluent A549-AT cells were pre-treated with different concentrations of amitriptyline (0 μM, 5 μM, 10 μM, 20 μM, or 30 μM) in MEM medium (Thermo Fisher Scientific) supplemented with 2% FCS (Biowest), 100 U/mL penicillin, and 0.1 mg/mL streptomycin (both Gibco) for 2 h at 37°C with 5% CO_2_. Afterwards, A549-AT cells were infected by adding 100 TCID_50_ of SARS-CoV-2 to the medium. Untreated A549-AT cells served as a negative control. After 2 days of incubation, the supernatants were harvested, and the viral titers were determined by endpoint dilution. For this, serial dilutions of the supernatants (1:10–1:10^8^) were incubated on A549-AT cells grown in 96-well microtiter plates. After 3 days of incubation (37°C, 5% CO_2_), the inoculation medium was removed, and the cells were stained with 0.5% crystal violet (Roth), dissolved in 20% methanol (Merck). The cells were then evaluated for cytopathic effects (CPEs) using light microscopy. The experiment was performed in triplicate.

### 2.6 Quantification of viral RNA

Cell culture supernatants were centrifuged to remove cell debris and stored at −80°C until further processing. For RNA purification from cell culture supernatants, the QIAamp Viral RNA Mini Kit (QIAGEN, Hilden, Germany) was used according to the manufacturer’s instructions, with prior DNase I digestion using the RNase-Free DNase Set (QIAGEN). Reverse transcription of 250 ng of total RNA was performed using the PrimeScript RT Master Mix (Takara, Kusatsu, Japan) to determine SARS-CoV-2 M- or N-gene expression. RT-qPCR was conducted with the GoTaq Probe qPCR Master Mix (Promega, Madison, WI, United States) following the manufacturer’s instructions, using gene-specific primers and probes (Thümmler et al., 2024). RT-qPCR was performed on a LightCycler 480 II (Roche, Basel, Switzerland) with an initial denaturation step at 95°C for 2 min (ramp rate: 4.4°C/s), followed by 40 cycles of denaturation at 95°C for 15 s (ramp rate: 4.4°C/s) and amplification at 60°C for 60 s (ramp rate: 2.2°C/s). The M- and N-genes were partially cloned into pCR2.1 (Thermo Fisher Scientific) or pMiniT 2.0 (NEB, Ipswich, MA, United States) to quantify M- and N-gene copy numbers. A 1:10 plasmid dilution series was used as a reference standard.

### 2.7 Cytotoxicity assay

The cytotoxic effects of different concentrations of amitriptyline on Vero E6 cells and A549-AT cells were evaluated using the Orangu^™^ cell counting solution (Cell Guidance Systems, Cambridge, United Kingdom) in accordance with the manufacturer’s guidelines. Amitriptyline solutions at concentrations of 0 μM, 5 μM, 10 μM, 20 μM, and 30 μM were prepared freshly in MEM (Thermo Fisher Scientific) supplemented with 2% FCS (Biowest), 100 U/mL penicillin, and 0.1 mg/mL streptomycin (Thermo Fisher Scientific). The different amitriptyline concentrations were added to A549-AT cells (1 × 10^4^ cells/well) in a 96-well plate and incubated at 37°C with 5% CO_2_. At three distinct time points (2 h, 24 h, and 48 h), the medium was replaced with fresh medium containing 10% Orangu^™^ solution and incubated for 60 min. Cell viability was determined by measuring absorbance at 450 nm using a Tristar 3 microplate reader (Berthold Technologies, Oak Ridge, TN, United States). Results were normalized against untreated control cells. The experiment was performed in triplicate.

### 2.8 Statistical analysis

Statistical analysis was performed using GraphPad Prism 10.0 software (San Diego, CA, United States). For the evaluation of categorical variables, a Kruskal–Wallis test (a non-parametric one-way ANOVA) was applied. Two-sided *p*-values <0.05 were considered significant.

## 3 Results

### 3.1 Initial investigation of the antiviral activity of amitriptyline against SARS-CoV-2

**Figure 1:**
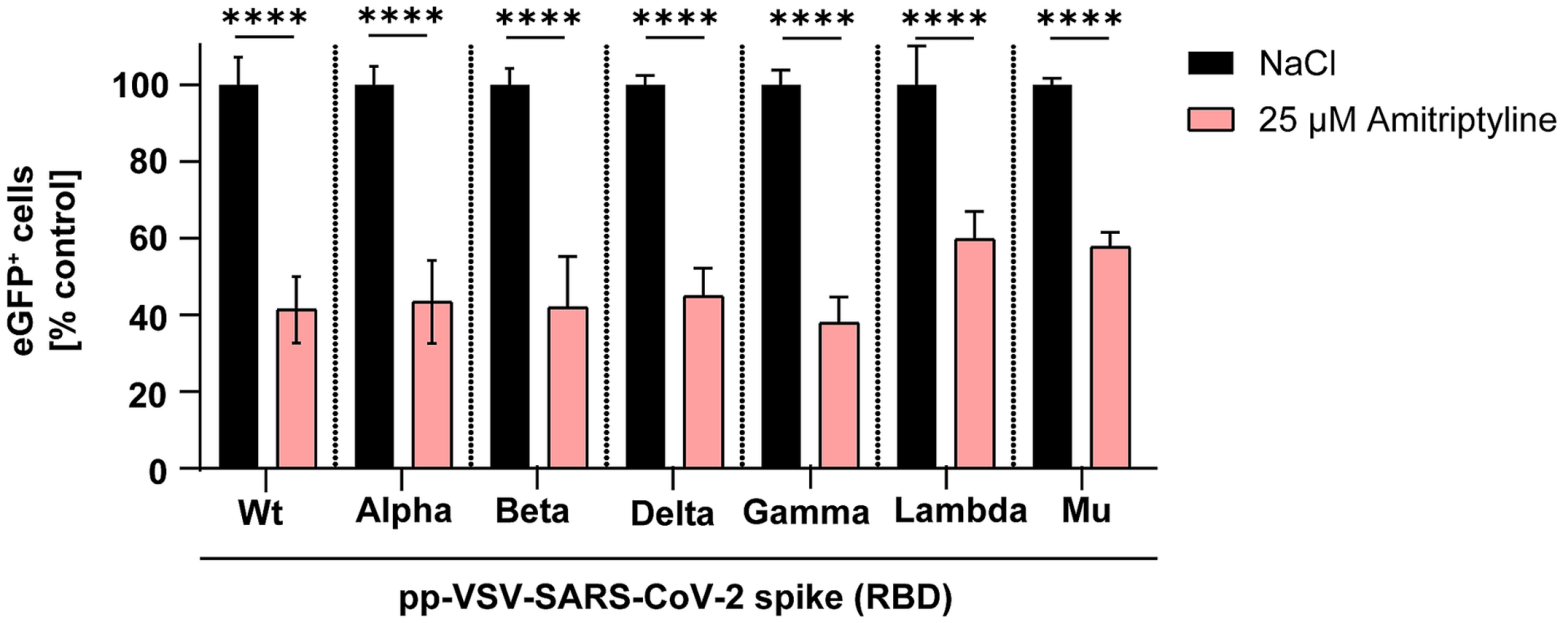
Suppression of SARS-CoV-2 infection using VSV-SARS-CoV-2 spike pseudotyped virus-like particles. Vero E6 cells were pre-treated with either NaCl (control, black column) or 25 μM amitriptyline (light red column) and then exposed to pp-VSV-SARS-CoV-2 spike carrying RBD mutations from SARS-CoV-2 variants. The effect of the antidepressant on cellular infection was assessed through quantitative measurement of eGFP expression, serving as a proxy for viral entry efficiency. Data represent mean values ± standard deviation (SD). Statistical differences between amitriptyline-treated samples and the NaCl control were determined using one-way ANOVA (^****^p < 0.0001).

First, the inhibitory effect of amitriptyline against SARS-CoV-2 infection was evaluated using pp-VSV-SARS-CoV-2 spike. Vero E6 cells were pre-treated with 25 μM amitriptyline for 4 h, followed by a 1-h infection with various pp-VSV-SARS-CoV-2 spike variations. The antiviral efficacy was assessed the following day using fluorescence microscopy and measured quantitatively via flow cytometry. Initial experiments utilized VSV pseudotyped virus-like particles carrying SARS-CoV-2 spike proteins with mutations exclusively in the receptor-binding domain (RBD). Amitriptyline treatment significantly reduced infection rates compared to the NaCl control for the wild type (*p* < 0.0001), as well as the Alpha (*p* < 0.0001), Beta (*p* < 0.0001), Delta (*p* < 0.0001), Gamma (*p* < 0.0001), Lambda (*p* < 0.0001), and Mu (*p* < 0.0001) variants. Compared to the respective NaCl-treated controls, the mean percentages of infected cells in amitriptyline-treated cultures were 41% for wild type, 43% for Alpha, 42% for Beta, 45% for Gamma, 38% for Delta, 60% for Lambda, and 58% for Mu (Figure 1). Amitriptyline significantly reduced infection rates across all variants.

**Figure 2:**
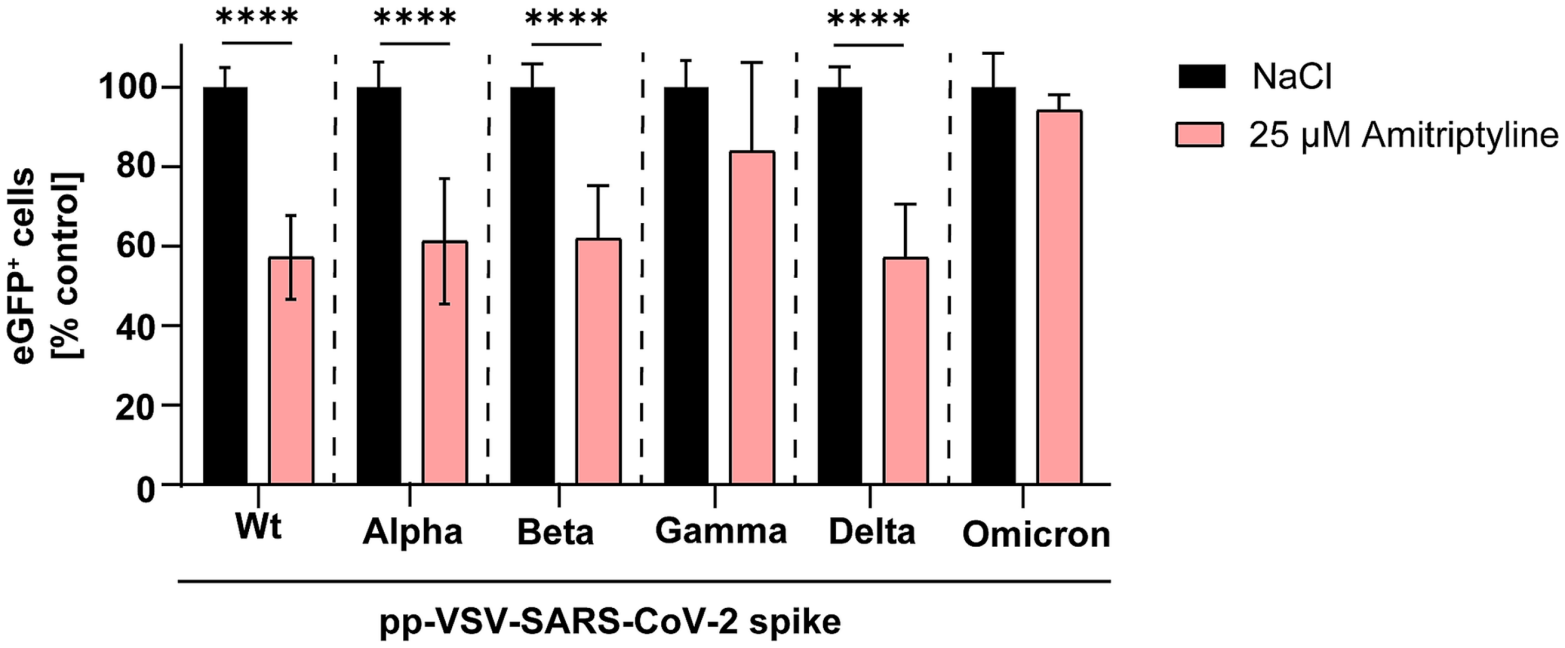
Inhibition of SARS-CoV-2 infection using distinct pp-VSV-SARS-COV-2 spike harboring all mutations of the spike variants. Vero E6 cells were pre-treated with NaCl (black column) or 25 μM amitriptyline (light red column) before being infected with pseudotyped virus-like particles carrying mutations from various SARS-CoV-2 variants. The effect of the antidepressant amitriptyline on cellular infection was assessed by quantifying eGFP expression using flow cytometry. Data are presented as the mean ± standard deviation (SD). Differences between the amitriptyline treatment and the NaCl control were analyzed using one-way ANOVA (^**^p < 0.01 and ^****^p < 0.0001).

Next, expression plasmids encoding spike proteins containing all mutations present in the respective variants of concern were utilized to generate pp-VSV-SARS-CoV-2 spike. The antiviral efficiency of amitriptyline against SARS-CoV-2 infection was evaluated by quantifying the number of GFP-positive cells. Amitriptyline significantly reduced infection rates of these pseudotyped virus-like particles carrying the wild-type D614G spike to 57% (*p* < 0.0001), Alpha to 61% (*p* < 0.0001), Beta to 62% (*p* < 0.0001), and Delta to 57% (*p* < 0.0001) compared to the NaCl control. There were no significant differences in infection rates for Gamma (reduction to 84%) and Omicron (reduction to 94%). Overall, amitriptyline demonstrated broad antiviral activity against pp-VSVSARS-CoV-2 spike representing distinct SARS-CoV-2 spike protein variants (Figure 2).

**Figure 3:**
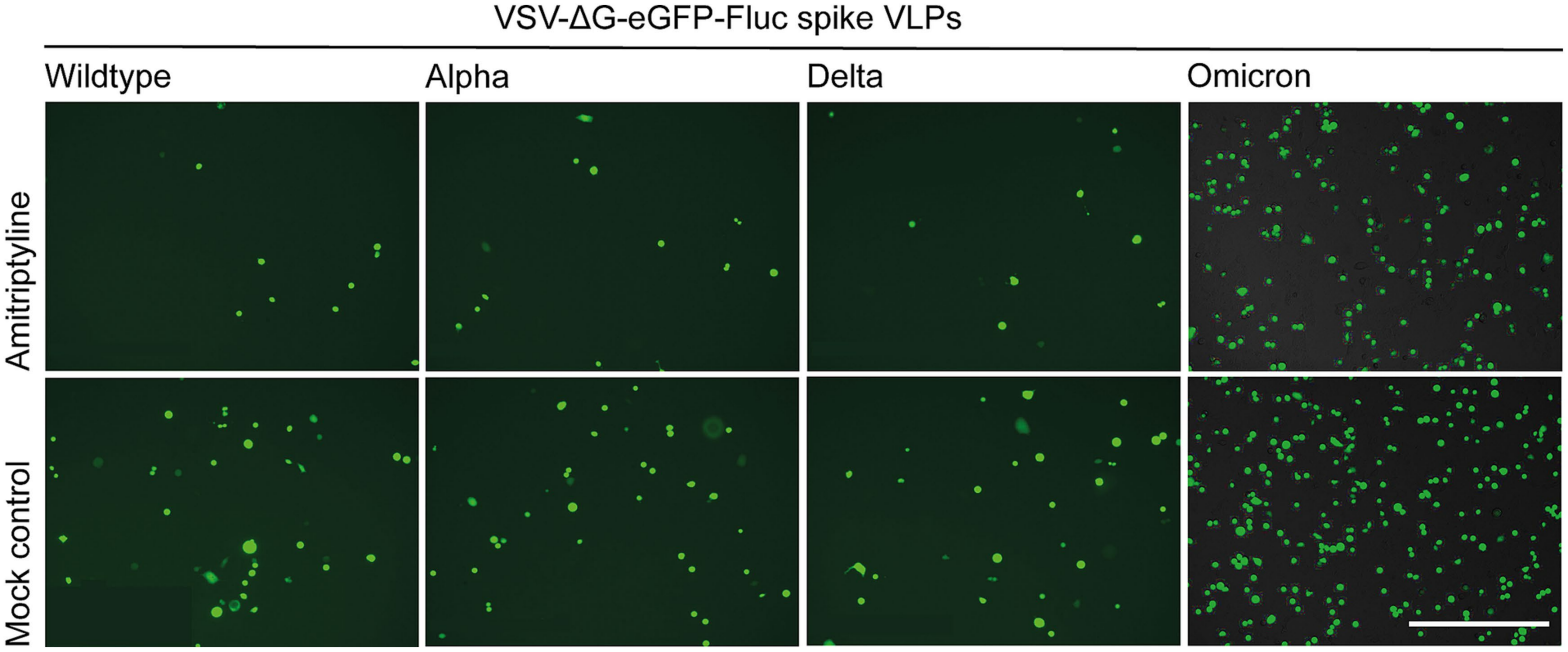
Qualitative assessment of the antiviral effects of amitriptyline against SARS-CoV-2. Vero E6 cells were pre-treated with NaCl or 25 μM amitriptyline and subsequently infected with pp-VSV-SARS-CoV-2 spike harboring all mutations from various SARS-CoV-2 variants. The impact of amitriptyline on the infection was analyzed using fluorescence microscopy. The scale bar indicates 400 μm.

Additionally, the inhibitory effect of amitriptyline on SARS-CoV-2 infection in Vero E6 cells was validated by fluorescence microscopy by using distinct pp-VSV-SARS-CoV-2 spike carrying spike proteins with full-length mutations of different variants (Figure 3).

### 3.2 Amitriptyline effectively blocks SARS-CoV-2 infection

**Figure 4:**
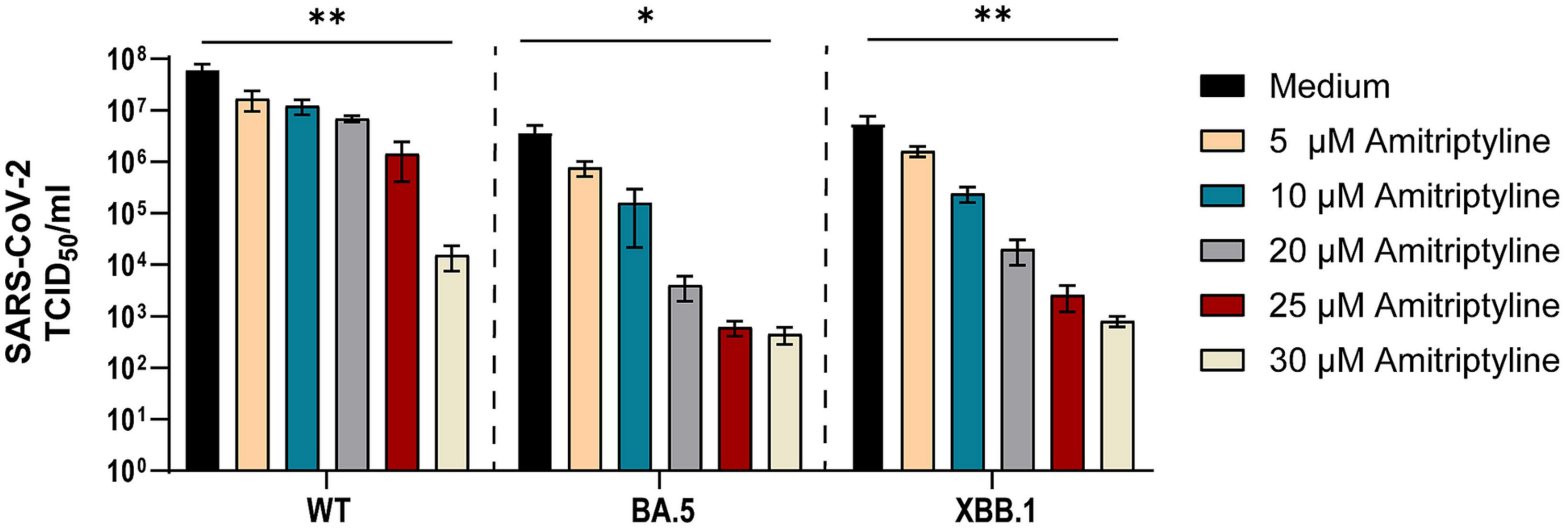
Potent neutralization of SARS-CoV-2 D614G, BA.5, and XBB.1 by amitriptyline. A549-AT cells were infected with 100 TCID_50_ of different clinical SARS-CoV-2 isolates (D614G, Omicron BA.5, and Omicron XBB.1) in the presence of various amitriptyline concentrations (0–30 μM). After 2 days of incubation, the viral loads in the respective cell culture supernatants were quantified using endpoint dilution. All experiments were performed in triplicate to ensure reproducibility. Data are shown as mean and standard deviation (SD). Differences between amitriptyline and the control were analyzed using Kruskal–Wallis test (^*^p < 0.05 and ^**^p < 0.01).

To analyze whether amitriptyline can inhibit viral replication or infection by authentic viruses, its antiviral efficacy was tested against clinical isolates of SARS-CoV-2, including D614G, Omicron BA.5, and Omicron XBB.1. Statistical comparisons were performed for all tested concentrations versus the medium control using one-way ANOVA with Dunnett’s multiple comparison test. Amitriptyline inhibited the replication of all tested SARS-CoV-2 variants in a concentration-dependent manner. At the highest tested concentration (30 μM), viral RNA levels were reduced by approximately 3–4 log10 for all three variants compared to the untreated control, with statistically significant effects observed for D614G (*p* = 0.0083), Omicron BA.5 (*p* = 0.0148), and Omicron XBB.1 (*p* = 0.0087). Lower concentrations (5–25 μM) resulted in stepwise reductions in viral load, ranging from ~1 log10 at 10 μM to ~2–3 log10 at 25 μM, although these effects did not reach statistical significance under the given conditions. These findings indicate a clear dose-dependent antiviral effect of amitriptyline (Figure 4).

**Figure 5:**
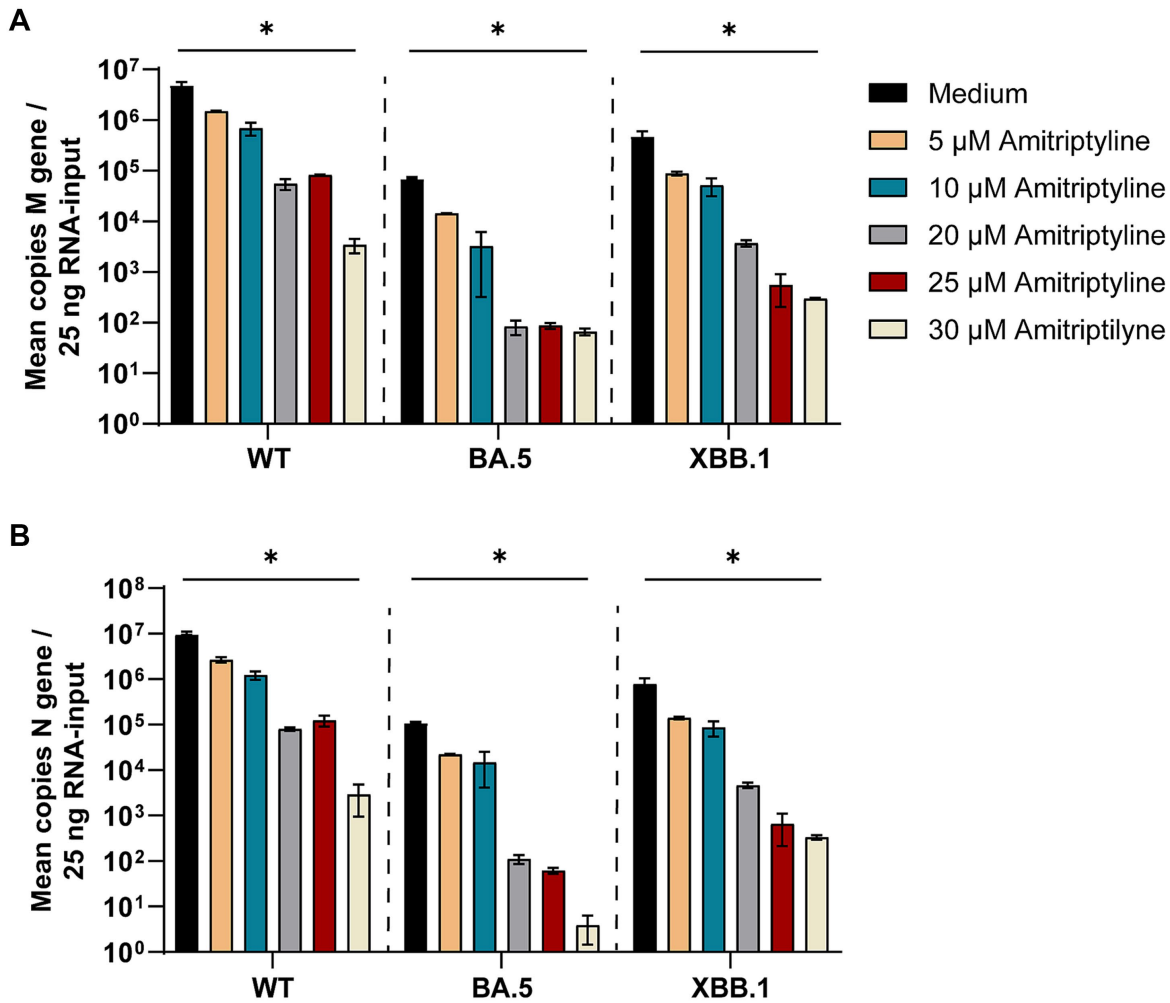
Treatment with amitriptyline significantly reduces SARS-CoV-2 M- and N-gene levels. A549-AT cells were infected with 100 TCID_50_ of different SARS-CoV-2 variants in the presence of varying concentrations of amitriptyline (0–30 μM). After 48 h of incubation, supernatants were harvested. Total RNA was isolated, reverse transcribed, and the SARS-CoV-2 M-gene **(A)** and N-gene **(B)** were quantified by RT-qPCR. Data are presented as the mean ± standard deviation (SD). Differences between amitriptyline-treated and mock-treated cell cultures were analyzed using Kruskal–Wallis test (^*^p < 0.05).

The antiviral efficacy of amitriptyline against SARS-CoV-2 variants was evaluated by quantifying viral RNA from cell culture supernatants. RNA isolation was followed by reverse transcription, and SARS-CoV-2 replication was assessed through RT-qPCR targeting the M gene (membrane protein) and N gene (nucleocapsid protein). Statistical analyses were conducted for each tested concentration in comparison to the medium control. Amitriptyline treatment induced a dose-dependent reduction in SARS-CoV-2 RNA copy numbers for both the M and N genes (Figure 5), consistent with the findings from endpoint dilution assays.

SARS-CoV-2 M-gene RNA levels were significantly reduced in all amitriptyline-treated cell cultures in a dose-dependent manner. A significant reduction in M-gene RNA levels was observed in all tested SARS-CoV-2 variants following treatment with amitriptyline at 30 μM for D614G (*p* = 0.0277), Omicron BA.5 (*p* = 0.0228) and Omicron XBB.1 (*p* = 0.0277) (Figure 5A). Accordingly, a significant reduction in N-gene RNA was observed after treatment with 30 μM amitriptyline in cell culture supernatants of SARS-CoV-2 D614G (*p* = 0.0277), Omicron BA.5 (*p* = 0.0237), and Omicron XBB.1 (*p* = 0.0277) (Figure 5B).

### 3.3 Amitriptyline showed no cytotoxic effects at SARS-CoV-2 neutralizing concentrations

**Figure 6:**
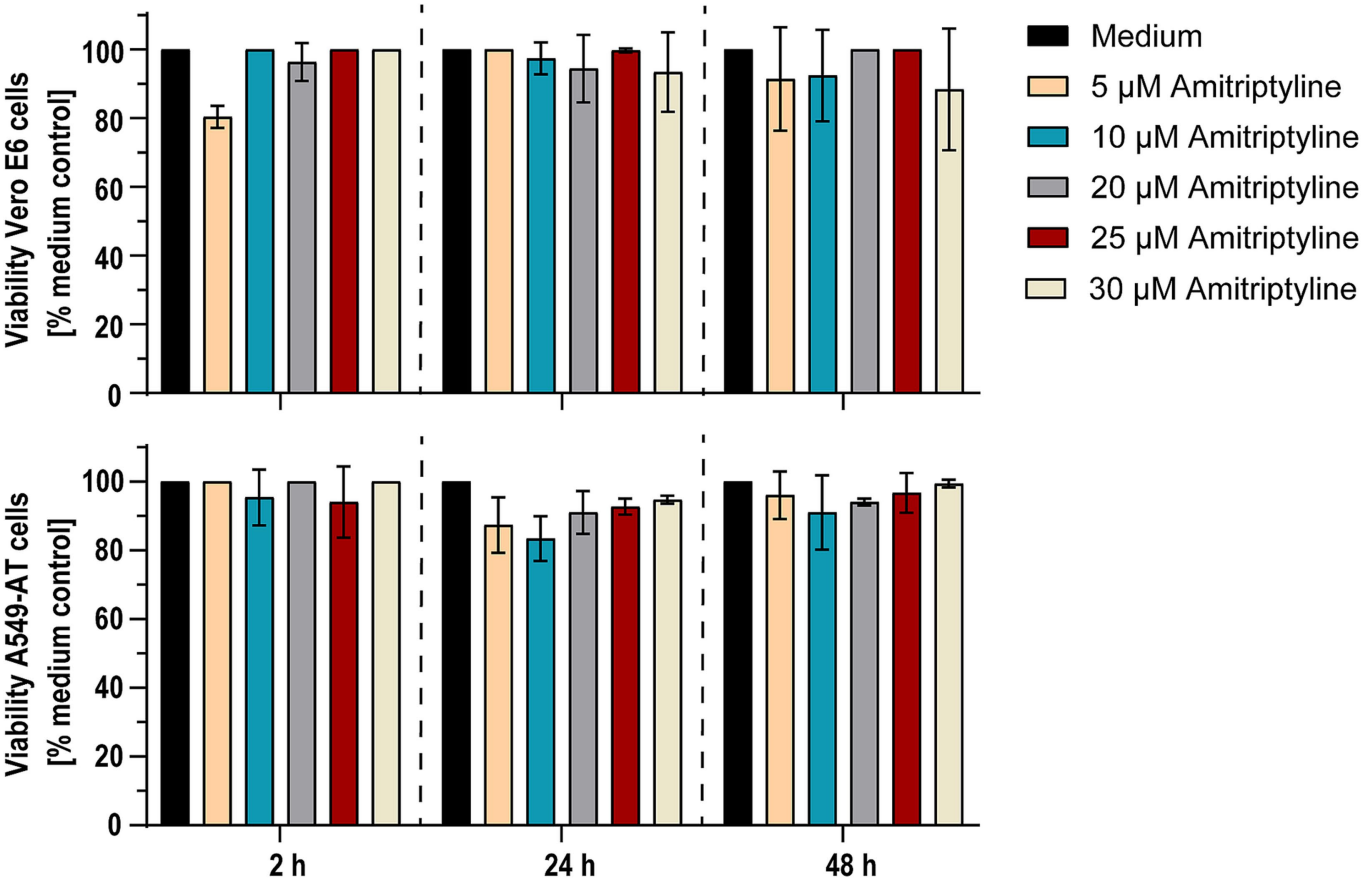
Amitriptyline showed no toxic effects on Vero E6 and A549-AT cells within 48 h. Potential cytotoxic effects of amitriptyline were assessed in Vero E6 (upper panel) and A549-AT cells (lower panel) using the Orangu^™^ cell counting solution. Various concentrations of amitriptyline were incubated with a confluent layer of Vero E6 or A549-AT cells, and cell viability was evaluated after 2, 24, and 48 h. All experiments were performed in triplicate. Data are presented as the mean ± standard deviation (SD). Differences between amitriptyline-treated cell cultures and mock treated cell cultures were analyzed using one-way ANOVA.

Possible cytotoxic effects of amitriptyline were examined by measuring cell viability after treating Vero E6 cells or A549-AT cells with various concentrations of amitriptyline (0–30 μM). After 2, 24, and 48 h of incubation at 37°C in 5% CO_2_, the cells were analyzed for viability. After 48 h, only subtoxic effects could be detected. Thus, amitriptyline effectively inhibited both the infection of Vero E6 cells with pp-VSV-SARS-CoV-2 spike virus-like particles and the infection of A549-AT cells with the SARS-CoV-2 variants D614G, Omicron BA.5, and XBB.1 at subtoxic concentrations (Figure 6).

## 4 Discussion

More than 4 years into the COVID-19 pandemic, SARS-CoV-2 continues to pose a significant global health burden, with ongoing hospitalizations, severe disease courses, and a substantial number of deaths. In addition, a considerable proportion of patients experience persistent symptoms collectively referred to as long COVID (Greenhalgh et al., 2024). As vaccination uptake declines and the number of breakthrough infections continues to rise, particularly with newly emerging viral variants, there is an urgent need for readily available therapeutic options to prevent or treat SARS-CoV-2 infections in a variant-independent manner (Chemaitelly et al., 2025; Raharinirina et al., 2025). Recent studies have identified several antidepressants as potential inhibitors of SARS-CoV-2 entry into host cells. Among these, fluoxetine and sertraline have demonstrated promising antiviral activity against early SARS-CoV-2 variants (Thümmler et al., 2024). These findings highlight the potential for repurposing existing, well-characterized drugs in the ongoing fight against COVID-19.

In the present study, we explored the antiviral effects of amitriptyline, another functional inhibitor of acid sphingomyelinase (FIASMA), against various clinical SARS-CoV-2 isolates, including D614G, Omicron BA.5, and Omicron XBB.1. To initially evaluate the antiviral potential of amitriptyline, we employed VSV-SARS-CoV-2 spike-pseudotyped virus-like particles bearing mutations either in the receptor-binding domain (RBD) or across the entire S1 subunit of the SARS-CoV-2 spike protein. This approach allows for rapid and efficient screening of candidate compounds under standard laboratory conditions, without the requirement for biosafety level 3 containment (Zettl et al., 2020; Hoffmann et al., 2021). Amitriptyline potently inhibited infection by all used VSV-SARS-CoV-2 spike-pseudotyped virus-like particles bearing either only RBD or all SARS-CoV-2 spike mutations, and clinical isolates of various SARS-CoV-2 variants, including D614G, Omicron BA.5, and Omicron XBB.1, at subtoxic concentrations of 10 μM to 30 μM. At a concentration of 30 μM amitriptyline, the viral load was reduced by two log levels for the D614G variant and by three log levels for the BA.5 and XBB.1 variants. These findings suggest that the neutralizing activity of amitriptyline against SARS-CoV-2 in cell culture is slightly lower compared to fluoxetine and sertraline, which we examined in a previous study. While amitriptyline demonstrated antiviral activity at concentrations of 10–30 μM, this effect appeared slightly less potent compared to fluoxetine and sertraline, which achieved complete viral neutralization at similar or lower concentrations in previous experiments (Thümmler et al., 2024). Direct EC₅₀ values under identical assay conditions are currently not available; thus, the comparative assessment remains semi-quantitative. The observed differences in potency may reflect variations in the degree of acid sphingomyelinase (ASM) inhibition, intracellular accumulation, or additional off-target effects. Fluoxetine and sertraline are known to inhibit ASM more potently than amitriptyline, and may exert additional antiviral mechanisms such as interference with endolysosomal trafficking (Kornhuber et al., 2010; Zimniak et al., 2021). The antiviral efficacy of amitriptyline against SARS-CoV-2 appears to be independent of the variant. This efficacy may be attributed to both direct and indirect interactions with the virus. Molecular docking studies suggest that amitriptyline can bind to both the spike (S) protein and the main protease (Mpro) of SARS-CoV-2. These interactions may impair the virus’s ability to attach to host cells and replicate. However, further experimental studies are needed to confirm these potential antiviral effects (Kutkat et al., 2022). It is important to note that the antiviral effects of amitriptyline differed slightly between pseudotyped virus-like particles carrying only RBD mutations, those bearing the full set of spike mutations characteristic of each variant, and authentic SARS-CoV-2 isolates. This discrepancy can be attributed to the inherent limitations of pseudotyped systems, which are designed to mimic only the viral entry process and do not replicate post-entry events such as genome replication, protein synthesis, or progeny virion assembly (Millet and Whittaker, 2016; Nie et al., 2020). In contrast, authentic SARS-CoV-2 completes the full replication cycle in host cells. This distinction is particularly relevant for drugs like amitriptyline, which may not only impair viral entry via inhibition of the acid sphingomyelinase/ceramide system, but could also interfere with intracellular processes involved in viral replication or egress (Schloer et al., 2020). As such, pseudotyped systems may underestimate the full antiviral potential of such compounds. While the antiviral effects of amitriptyline were observed at concentrations of 10 to 30 μM, which exceed typical plasma levels (0.3 to 1.2 μM), its pharmacokinetic profile supports the potential for clinically relevant tissue exposure. Amitriptyline is a highly lipophilic compound with extensive tissue binding and a large volume of distribution, enabling significant accumulation in peripheral organs including the lungs, the primary site of SARS-CoV-2 replication (Gupta et al., 1999; Gillman, 2007). Consequently, local concentrations in lung tissue may reach or exceed those required for antiviral efficacy observed *in vitro*. Nevertheless, further *in vivo* studies and tissue distribution analyses are necessary to substantiate this hypothesis and determine whether effective drug levels are achieved at the site of infection. Furthermore, considering the pulmonary tropism of SARS-CoV-2, alternative administration routes such as inhaled formulations may offer a promising strategy to enhance local drug concentrations in the lungs while minimizing systemic exposure and related side effects (Patton and Byron, 2007; Darquenne, 2012). Inhaled delivery has been successfully utilized for other respiratory therapeutics and may represent a viable approach for repurposing amitriptyline in the context of COVID-19. However, dedicated preclinical studies are required to evaluate the feasibility, pharmacodynamics, and safety of this route of administration. Similar to fluoxetine and sertraline, amitriptyline acts as a FIASMA. Acid sphingomyelinase (ASM) catalyzes the conversion of sphingomyelin into ceramide, a lipid that facilitates viral entry by forming ceramide-enriched membrane domains. By inhibiting ASM, amitriptyline avoids increasing ceramide levels on the cell surface, thereby preventing the formation of these domains and therefore SARS-CoV-2 from entering epithelial cells. This mechanism has been demonstrated in both *in vitro* and *ex vivo* studies using early SARS-CoV-2 variants, including experiments with human nasal epithelial cells treated with amitriptyline (Asadi Anar et al., 2022). Building on previous research, our findings suggest that early use of fluoxetine, sertraline, or amitriptyline in SARS-CoV-2 infections involving recent and emerging variants may lower the risk of severe disease progression or death (Hoertel et al., 2023). A systematic review encompassing various antidepressants, including amitriptyline, identified studies on antidepressant use in COVID-19 treatment. However, the clinical data predominantly focused on selective serotonin reuptake inhibitors (SSRIs) like fluvoxamine and fluoxetine, which have shown more consistent evidence in reducing COVID-19 severity. Amitriptyline-specific clinical data remain limited and inconclusive (Zheng et al., 2022). Our study has some limitations that should be acknowledged. The antiviral effects of amitriptyline were evaluated using the A549-AT cell line, which was specifically engineered to allow robust replication of all major SARS-CoV-2 variants, including BA.5 and XBB.1. This represents a clear advantage over other commonly used models such as Vero E6 cells, which exhibit limited permissiveness to recent SARS-CoV-2 lineages due to their deficient expression of relevant entry factors. However, the use of immortalized cell lines may not fully replicate the cellular complexity of the human respiratory tract. While primary human bronchial or nasal epithelial cells represent more physiologically relevant models, their susceptibility to SARS-CoV-2 infection can vary substantially depending on donor, culture conditions, and viral variant. Therefore, for future preclinical development of amitriptyline as a potential antiviral agent, it will be important to confirm our findings in primary airway epithelial cells and in animal models, to better assess pharmacodynamic parameters and *in vivo* efficacy. The antiviral effect size observed in this study is broadly consistent with previous findings on amitriptyline. In earlier studies, amitriptyline showed inhibitory activity against early SARS-CoV-2 isolates *in vitro* at comparable concentrations (Schloer et al., 2020). Our data extend these observations to include recent variants of concern such as Omicron BA.5 and XBB.1. However, in direct comparison, the antiviral potency of amitriptyline appears to be slightly lower than that of other FIASMAs like fluoxetine and sertraline, which achieved complete viral neutralization at similar or even lower concentrations in comparable assay systems (Thümmler et al., 2024). These differences in potency should be considered when evaluating the clinical potential of individual FIASMAs. While amitriptyline is generally considered safe when used within its therapeutic range, its anticholinergic and cardiotoxic side effects, particularly at higher doses, must be carefully considered, especially in elderly patients or individuals with preexisting cardiovascular conditions (Boyer and Shannon, 2005; Gillman, 2007). These patient groups often overlap with populations at increased risk for severe COVID19. Therefore, any future clinical application of amitriptyline as an antiviral agent would require careful dose optimization and risk benefit evaluation tailored to the specific patient population.

In conclusion, this study demonstrates that amitriptyline exhibits potent antiviral activity against SARS-CoV-2 in a variant-independent manner. While amitriptyline has demonstrated potential antiviral mechanisms against SARS-CoV-2 in laboratory studies—primarily through the inhibition of acid sphingomyelinase and subsequent reduction of ceramide-mediated viral entry—there is currently insufficient clinical evidence to support its efficacy in treating COVID-19 patients. Further research is warranted to assess its therapeutic potential, including clinical trials to evaluate its safety and efficacy. Additionally, investigations into possible synergistic effects with other antiviral or anti-inflammatory agents, as well as its applicability in both prophylactic and therapeutic settings, are essential to determine its clinical relevance in COVID-19 management.

## Data Availability

The raw data supporting the conclusions of this article will be made available by the authors, without undue reservation.
